# MiR-27a as a predictor for the activation of hepatic stellate cells and hepatitis B virus-induced liver cirrhosis

**DOI:** 10.18632/oncotarget.23262

**Published:** 2017-12-15

**Authors:** Hui Zhang, Xiu-Li Yan, Xin-Xin Guo, Miao-Juan Shi, Yi-Yu Lu, Qian-Mei Zhou, Qi-Long Chen, Yi-Yang Hu, Lie-Ming Xu, Shuang Huang, Shi-Bing Su

**Affiliations:** ^1^ Research Center for Traditional Chinese Medicine Complexity System, Shanghai University of Traditional Chinese Medicine, Pudong, Shanghai 201203, China; ^2^ Yueyang Hospital of Integrated Traditional Chinese and Western Medicine, Shanghai University of Traditional Chinese Medicine, Hongkou, Shanghai 200437, China; ^3^ Shanghai Shuguang Hospital, Shanghai University of Traditional Chinese Medicine, Pudong, Shanghai 201203, China; ^4^ Department of Anatomy and Cell Biology, University of Florida College of Medicine, Gainesville, FL, USA

**Keywords:** MiR-27a, predictor, hepatic stellate cells, hepatitis B virus-induced liver cirrhosis

## Abstract

Circulating microRNAs (miRNAs) can be employed as biomarkers to diagnose liver and other diseases. Noninvasive approaches are needed to complement and improve the current strategies for screening for biomarkers liver cirrhosis. We determined whether the serum levels of miRNAs can distinguish between chronic hepatitis B (CHB) and CHB-induced cirrhosis (HBC) and investigated the potential mechanisms involved. We found that serum miR-27a was significantly up-regulated in HBC, distinguishing HBC from CHB and healthy controls (Ctrl) (*P*<0.0001, the area of under the curve (AUC) =0.82 and 0.87, respectively). Specifically, when miR-27a was combined with miR-122, HBC was differentiated from CHB with an AUC=0.94. The serum miR-27a level in HBC patients with hepatic decompensation was significantly higher than that in patients with compensated HBC (*P*=0.0009). MiR-27a was also significantly up-regulated in the serum of rats with DMN-induced liver cirrhosis compared to that in saline-treated rats (*P*<0.0001). Furthermore, the down-regulation of miR-27a inhibited the proliferation and overexpression of miR-27a in activated hepatic stellate cells (HSCs) through the up-regulation of α-SMA and COL1A2 expression by targeting PPARγ, FOXO1, APC, P53 and RXRα. Our study demonstrated that circulating miR-27a can be used as a predictor for the activation of HSCs and the occurrence and development of HBC.

## INTRODUCTION

Liver cirrhosis, one of the most common non-neoplastic causes of mortality worldwide, is characterized by the replacement of liver tissue by fibrosis (scar tissue) and regenerative nodules (lumps that result from the attempted repair of damaged tissue) [1]. The principal causes of this disease include chronic viral infection, excess alcohol consumption, metabolic syndrome, and autoimmune disorders. Chronic hepatitis B (CHB) is a major cause of liver cirrhosis (HBC) in China [2, 3]. Up to 40% of patients with CHB progress to chronic end-stage liver disease or hepatocellular carcinoma (HCC) during their lifetime [4].

Liver biopsy has traditionally been considered the gold standard for diagnosing cirrhosis [5]. However, this invasive technique has some limitations, including morbidity and mortality, observer variability, and sampling variation [6, 7]. Therefore, finding an effective method and reliable biomarkers to ensure an early diagnosis would play a pivotal role in improving the treatment and prognosis of HBC.

MicroRNAs (miRNAs), which are evolutionarily conserved, are small (typically 22 nt in size) regulatory RNA molecules that modulate the levels of specific targets; therefore, they are actively involved in a wide range of physiologic and pathologic processes [8, 9]. Numerous studies have shown that miRNAs are very stable in circulation systems, and tissue or organ-specific intracellular miRNAs can often be detected in the blood under pathological conditions [10–12]. Circulating miRNAs have been reported to be promising biomarkers for various pathologic conditions, including liver disease. Some miRNAs have been demonstrated to change after the initiation and progression of various liver diseases, such as CHB [13, 14], HBC [15], and HCC [16].

The objective of this study was to investigate the potential of certain serum miRNAs for use as novel non-invasive biomarkers for the early diagnosis of HBC. We performed serum miRNA profiling using microarrays in a set of HBC and CHB cases, identifying miRNAs that could be used to detect HBC. Validation in an independent cohort of individuals using quantitative RT-PCR (RT-qPCR) allowed us to confirm that the identified miR-27a was significantly up-regulated in the serum of patients with HBC or rats with DMN-induced liver cirrhosis, TGFβ1-activated hepatic stellate cells(HSCs) and the culture medium. Subsequently, we investigated the profibrogenic effects and associated mechanisms of the activation of HSCs by miR-27a. Our study demonstrated that circulating miR-27a could be a potential predictor for HSCs activation and the occurrence and development ofHBC.

## RESULTS

### Expression profiling in CHB and HBC serum

To assess the differential circulating miRNA expression profiles between HBC and CHB, miRNA microarray experiments were conducted on the total RNA obtained from serum samples from 10 HBC and 10 CHB cases. Among the 851 miRNAs analyzed, 38 were differentially expressed between HBC and CHB. Compared with CHB patients, 33 miRNAs were up-regulated and 5 miRNAs were down-regulated in HBC patients (fold-change>2.0 and *P*-value<0.01) (Table 1).

**Table 1:** Differentially expressed miRNAs in HBC and CHB. CHB: chronic hepatitis B; HBC: CHB-induced cirrhosis

miRNA	Fold-change (HBC/CHB)	*P*-values
miR-146a	5.72	2.07E-03
miR-221	5.23	9.33E-03
**miR-151-5p**	5.09	7.72E-04
miR-199a-3p	4.69	1.28E-03
miR-130a	4.51	7.00E-03
**miR-27a**	4.24	2.78E-03
miR-199a-5p	4.18	9.15E-03
miR-103	4.08	3.92E-03
**miR-27b**	3.86	1.78E-03
**miR-142-3p**	3.79	1.45E-03
miR-21	3.51	4.02E-03
miR-324-5p	3.47	5.46E-03
miR-23b	3.38	2.01E-03
miR-140-5p	3.29	3.26E-04
miR-148b	3.28	6.96E-03
miR-652	3.16	4.48E-03
miR-340	3.14	8.99E-03
miR-338-3p	3.12	1.76E-03
miR-126	3.03	1.41E-03
miR-331-3p	2.89	2.77E-03
miR-23a	2.73	1.77E-03
miR-30b	2.71	5.44E-03
miR-374b	2.69	3.19E-03
miR-301a	2.67	7.68E-03
miR-223	2.63	4.42E-03
miR-33a	2.58	6.51E-03
miR-30c	2.52	3.81E-03
miR-148a	2.49	7.31E-03
miR-744	2.47	8.45E-03
**miR-424**	2.24	9.77E-04
miR-17	2.2	3.26E-03
miR-128	2.08	3.82E-03
miR-30e^*^	2.06	6.78E-03
miR-939	0.49	6.52E-03
miR-1275	0.42	8.86E-03
miR-1915	0.35	5.49E-03
miR-638	0.34	7.53E-03
miR-940	0.31	5.65E-03

### Validation of serum miRNA expression by RT-qPCR

**Figure 1:**
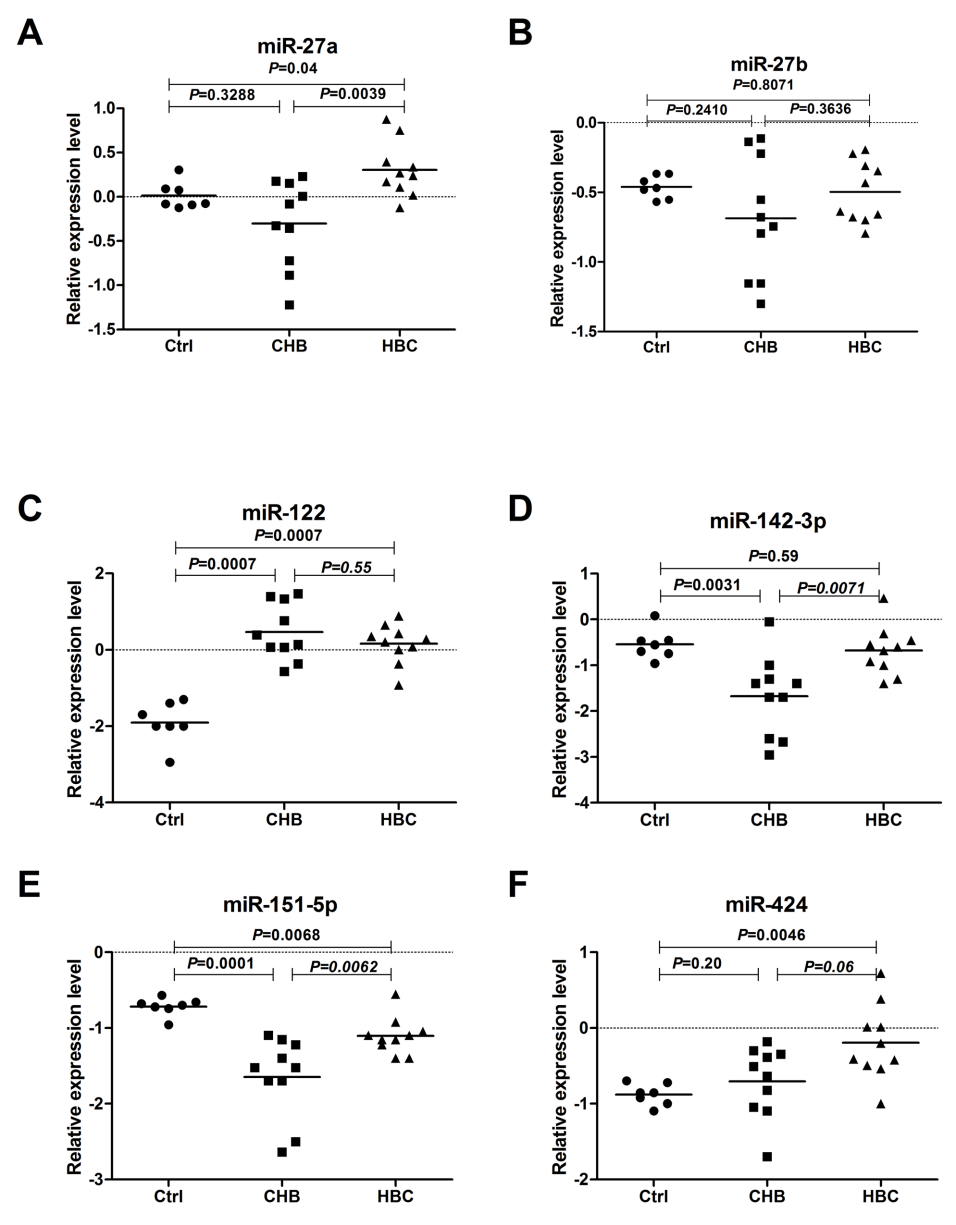
Serum levels of miRNAs in CHB, HBC, and Ctrl subjects The levels of serum miR-27a **(A)**, miR-27b **(B)**, miR-122 **(C)**, miR-142-3p **(D)**, miR-151-5p **(E)** and miR-424 **(F)** in CHB (n=10), HBC (n=10) and Ctrl (n=7) subjectswere measured by RT-qPCR. The line for each group represents the median value of the indicated miRNA. The values were normalized to miR-24 and are shown in log10 scale on the Y-axis. CHB: chronic hepatitis B; HBC: CHB-induced cirrhosis; Ctrl: healthy control.

Initially, we performed RT-qPCR to confirm the microarray results in 27 randomly selected samples from 10 HBC cases, 10 CHB cases, and 7 healthy controls (Ctrl). For these experiments, 5 candidate miRNAs (miR-27a, miR-27b, miR-142-3p, miR-151-5p, and miR-424) were chosen because they were among in the 38 deregulated miRNAs in HBC compared with CHB. MiR-122 was also detected, which has been described as a liver-specific miRNA that exhibits an excellent correlation with hepatitis B virus infection, cholesterol metabolism and HCC [11, 13, 14]. Overall, the RT-qPCR and microarray results were correlated, except for those for miR-27b and miR-424, which were found to be up-regulated to a lesser degree by RT-qPCR (Figure 1A–1F).

### Serum miR-27a level was up-regulated in HBC

**Figure 2:**
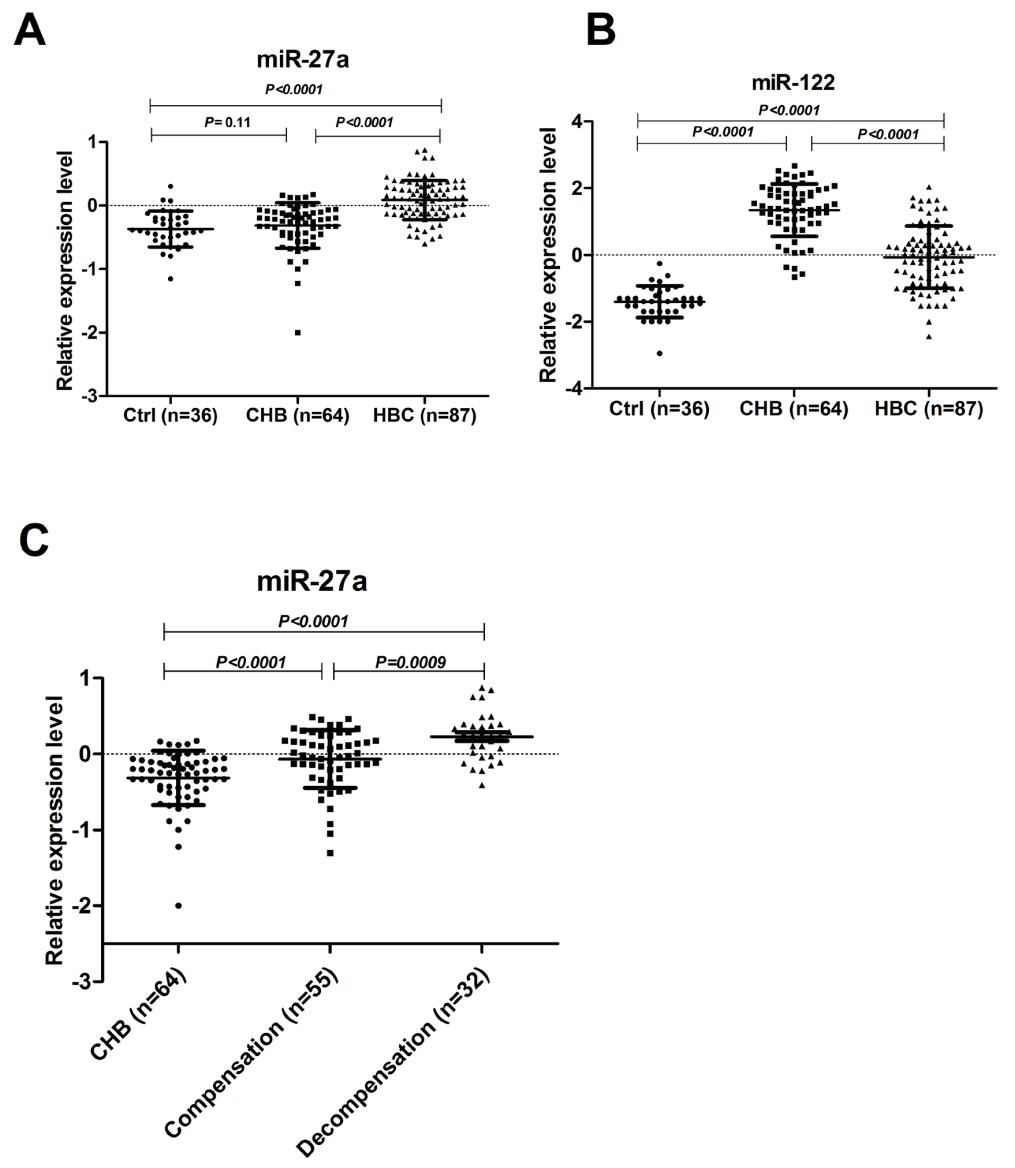
Serum levels of miR-27a and miR-122 in CHB, HBC and Ctrl subjects The levels of serum miR-27a **(A)** and miR-122 **(B)** in HBC (n=87), CHB (n=64), and Ctrl (n=36) subjects were measured by RT-qPCR. The serum miR-27a levels **(C)** in patients with (n=32) and without hepatic decompensation (n=55) were measured. The line for each group represents the median value of the indicated miRNA. The values were normalized to miR-24 and are shown in log10 scale on the Y-axis. CHB: chronic hepatitis B; HBC: CHB-induced cirrhosis; Ctrl: healthy control.

**Figure 3:**
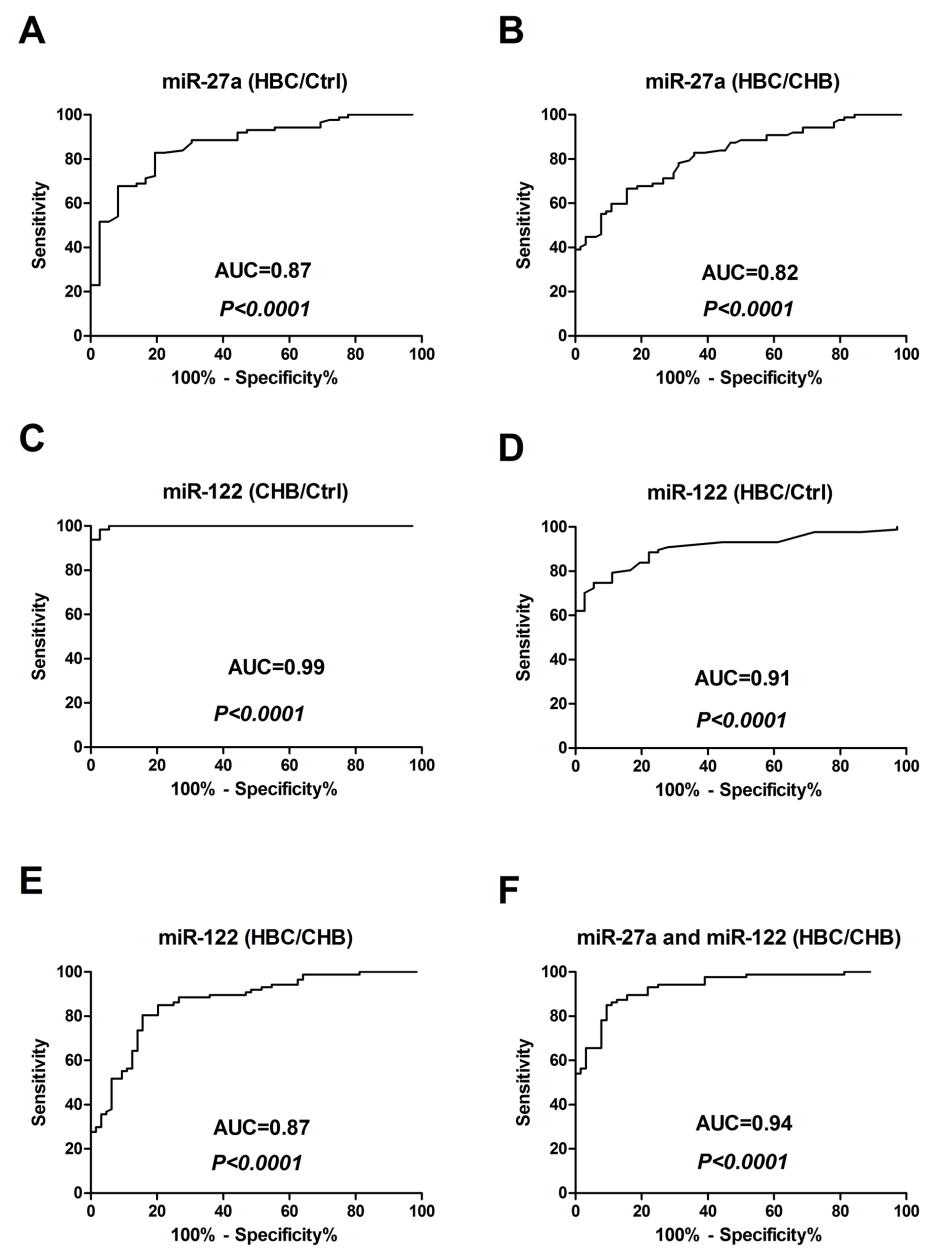
Receiver-operating characteristic (ROC) curves for miR-27a and miR-122 to discriminate between HBC subjects and Ctrl and CHB subjects **(A)** miR-27a (HBC/Ctrl); **(B)** miR-27a (HBC/CHB); **(C)** miR-122 (CHB/Ctrl); **(D)** miR-122 (HBC/Ctrl); **(E)** miR-122 (HBC/CHB); and **(F)** miR-27a and miR-122 (HBC/CHB). CHB: chronic hepatitis B; HBC: CHB-induced cirrhosis; Ctrl: healthy control; and AUC: area under the ROC curve.

To determine whether serum miR-27a was over-expressed in HBC patients, miR-27a and miR-122 were identified as candidates for further testing via RT-qPCR of samples from 87 HBC, 64 CHB, and 36 Ctrl subjects. Serum miR-27a was significantly up-regulated in HBC, which helped differentiate HBC from CHB and Ctrl samples (*P*<0.0001 for both) (Figure 2A). The serum miR-122 level was significantly higher in the HBC and CHB groups than in the Ctrl group (*P*<0.0001 for both). The serum miR-122 level was also markedly higher in the CHB group than in the HBC group (*P*<0.0001). The miR-27a level in the serum of patients with and without hepatic decompensation was measured. The serum miR-27a level in patients with hepatic decompensation was significantly higher compared to that in patients with compensated HBC (*P*=0.0009) (Figure 2C). On the other hand, miR-27a showed a good capacity to discriminate between the groups. Comparing HBC subjects with CHB and Ctrl subjects, the ROC curve areas of miR-27a were 0.82 (95% CI: 0.75-0.88) (Figure 3A) and 0.87 (95% CI: 0.80-0.93) (Figure 3B), respectively. The sensitivity and specificity values of miR-27a were 82.8% and 80.6% in the HBC and Ctrl subjects, respectively, and the sensitivity and specificity were 66.7% and 84.4% in the HBC subjects and CHB subjects, respectively. The ROC curve area for the combination of miR-27a and miR-122 was 0.94 (Figure 3F). These results demonstrate that the level of miR-27a may distinguish HBC from CHB and Ctrl cases.

### Serum miR-27a expression was up-regulated in rat models of DMN-induced liver cirrhosis

**Figure 4:**
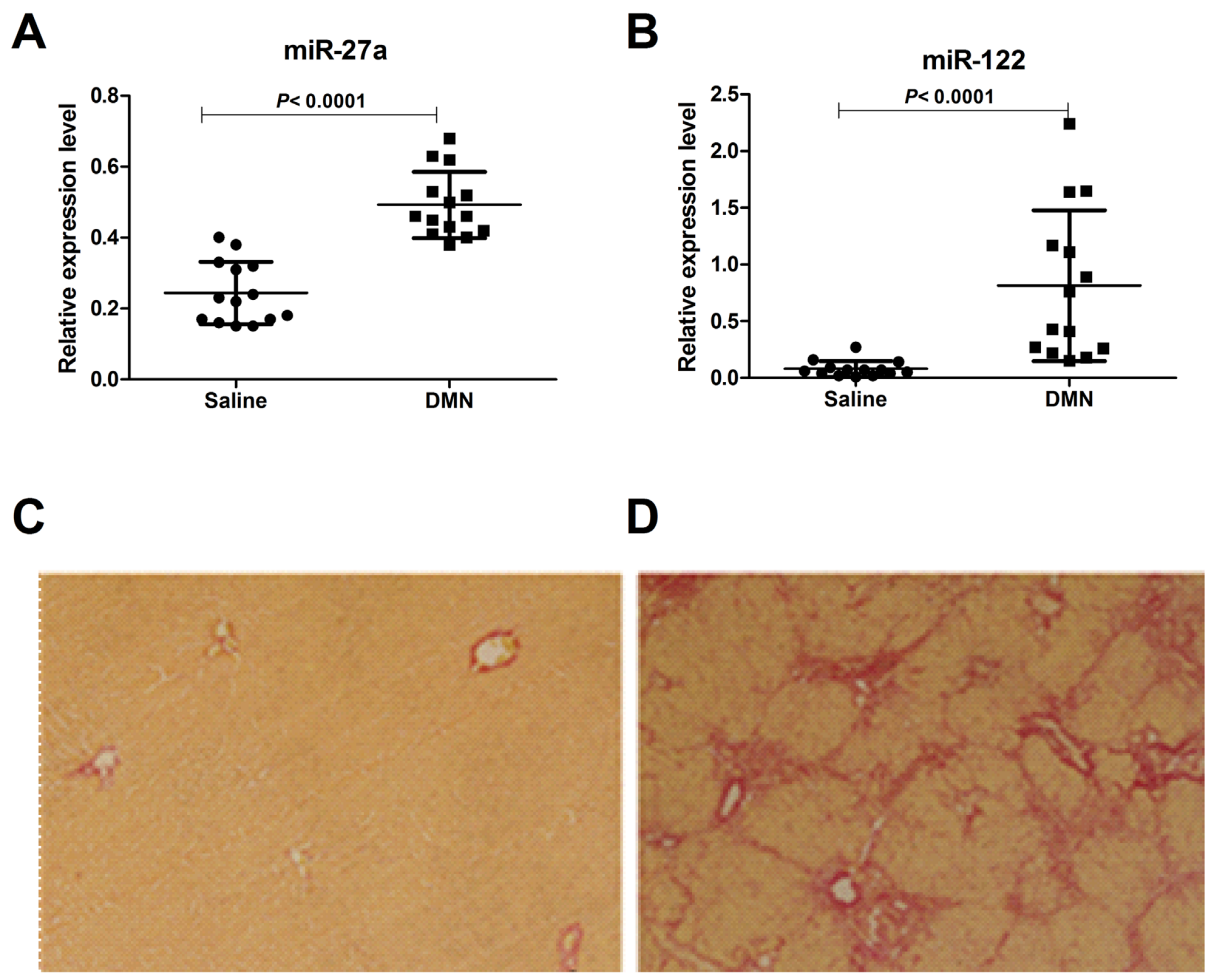
Serum levels of miR-27a and miR-122 in a rat model of liver cirrhosis induced by DMN and saline The serum miR-27a **(A)** and miR-122 **(B)** levels in rats with cirrhosis (n=14) and saline-treated rats (n=14) were measured by RT-qPCR. The values were normalized to miR-24. **(C, D)** Paraffin-embedded liver tissue sections were stained in 1% Sirius red, revealing the collagen distribution (×100). **(C)** Normal liver with collagen staining mainly in the portal spaces. **(D)** Fibrotic liver after 4 weeks of DMN treatment showing abnormal collagen accumulation in the portal areas and the formation of collagen-rich septa between hepatic lobules with proliferation of the bile ducts.

To further assess whether differential miR-27a production was associated with rodent models of liver cirrhosis, we used a rat model of DMN-induced liver cirrhosis. After 4 weeks of DMN treatment, the miR-27a and miR-122 concentrations in sera were significantly up-regulated compared to those of normal animals (*P*<0.0001) (Figure 4A and 4B). Liver histopathologic evaluations revealed features of morphological changes, including hepatic steatosis (Figure 4C and 4D). The mRNA and protein levels of α-SMA and COL1A2 were significantly up-regulated in the tissue of rats with liver cirrhosis compared to those in normal animals (Supplementary Figure 1).

### MiR-27a was induced by TGFβ1 in LX2 cells and in the culture medium

**Figure 5:**
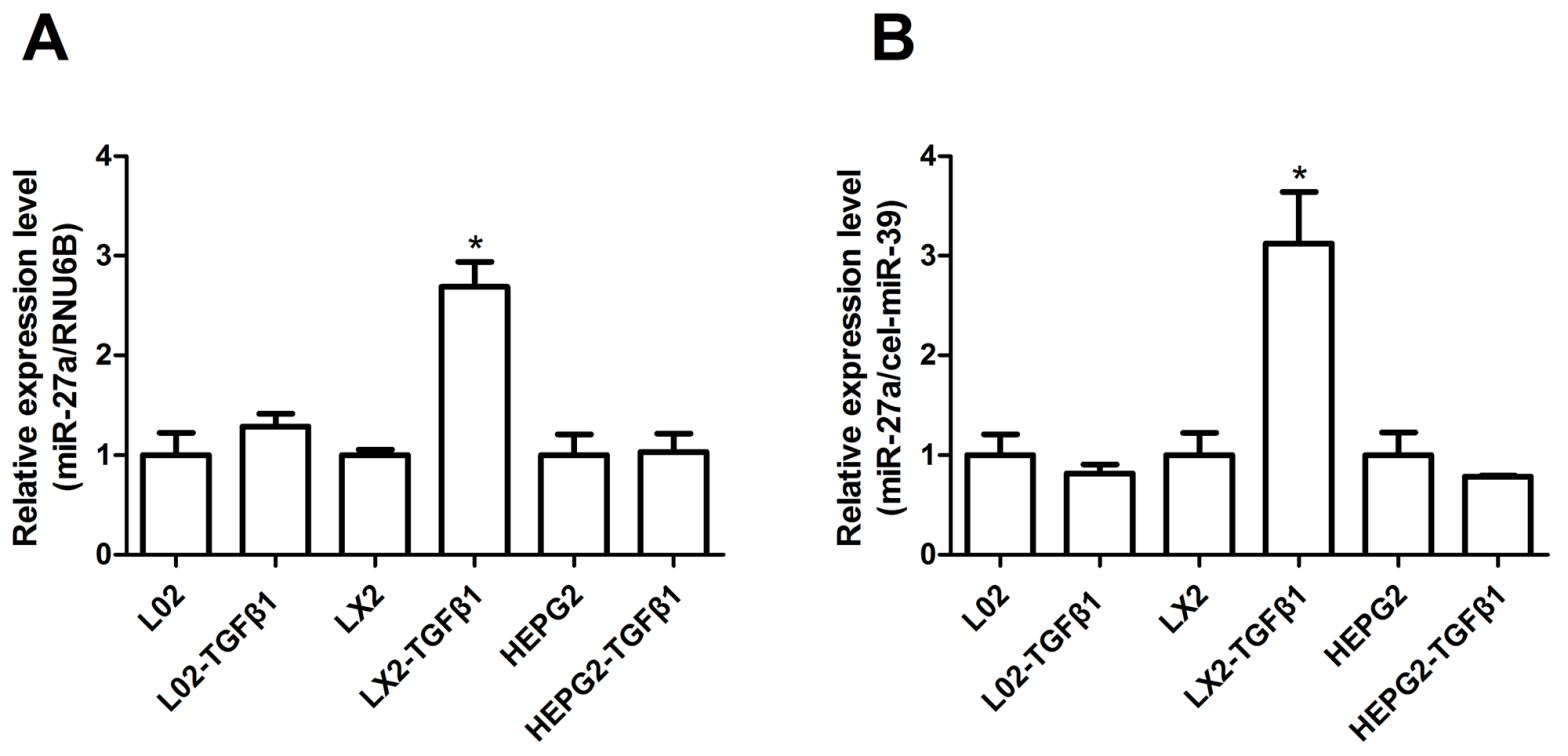
miR-27a expression was up-regulated in response to TGFβ1 stimulation in LX2 cells and in the culture medium The relative miR-27a expression in L02, LX2, and HepG2 cells **(A)** and in the culture medium **(B)** was measured by RT-qPCR after treatment with 10 ng/ml TGFβ1 for 24 h. The results are expressed as the mean; error bars denote the standard error of the mean. ^*^P<0.01. Relative expression was normalized by RNU6B for cells and cel-miR-39 for the culture medium.

Based on the prominent up-regulation of circulating miR-27a in HBC patients, we hypothesized that miR-27a might be centrally involved in cellular responses to profibrogenic signals. The most relevant collagen-producing cell types in liver fibrosis are HSCs, and cytokine transforming growth factor-β (TGFβ) is one of the key profibrogenic mediators. We assessed the stimulatory effect of TGFβ1 on miR-27a expression in LX2 (a human HSC line), L02, and HepG2 cells and in the culture medium. When human LX2 cells were stimulated with recombinant TGFβ1 (10 ng/ml), miR-27a expression was substantially up-regulated in LX2 cells and in the culture medium (Figure 5A and 5B). However, miR-27a did not exhibit specific changes following TGFβ1 stimulation in L02 and HEPG2 cells and in the culture medium. These data support that miR-27a may be associated with the regulation of hepatic fibrogenesis.

### Inhibition of miR-27a attenuates HSCs proliferation and inhibits TGFβ-induced expression of fibrosis-related genes

**Figure 6:**
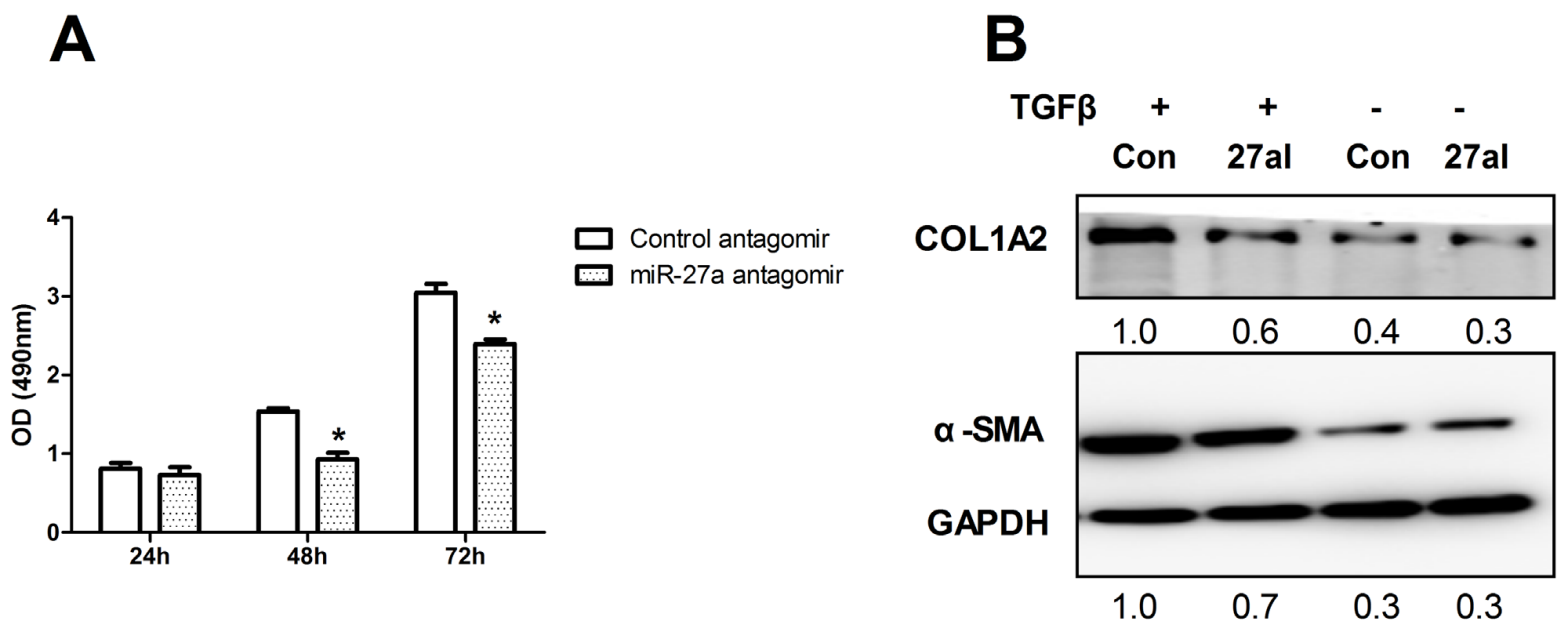
Inhibition of miR-27a attenuates HSCs proliferation and inhibits TGFβ-induced expression of fibrosis-related genes **(A)** LX2 cells were transfected with 100 nM miR-27a antagomir and a control antagomir. Cell proliferation rates were determined using the MTS assay. The results are expressed as the mean; error bars denote the standard error of the mean. ^*^P<0.01. **(B)** Inhibition of miR-27a expression attenuated the TGFβ-induced expression of α-SMA and COL1A2 in LX2 cells. LX2 cells were transfected with negative control (Con) or miR-27a antagomir (27I, 100 nM) for 48 hours, followed by stimulation with10 ng/ml TGFβ (+) or remained untreated (-) for 24 hours before immunoblotting. The intensity of each band was densitometrically quantified. The α-SMA and COL1A2 level in each sample was normalized by that of GAPDH (internal control).

To determine the role of miR-27a in HSCs proliferation, we next examined LX2 cell viability. The proliferation of miR-27a antagomir-transfected LX2 cells was significantly inhibited compared to that of negative control cells (Figure 6A). The level of miR-27a significantly increased when LX2 cells were activated by TGFβ1 treatment (Figure 5A). Furthermore, the expression of α-SMA and COL1A2, markers of fibrogenic cell activation, was up-regulated in TGFβ1-treated LX2 cells (Figure 6B), but this effect was significantly reduced by the inhibition of miR-27a expression (Figure 6B). These results suggest that miR-27a up-regulation may facilitate the TGFβ-induced activation of HSCs.

### miR-27a targeted PPARγ and inhibited PPARγ-induced up-regulation of fibrosis-related gene expression

**Figure 7:**
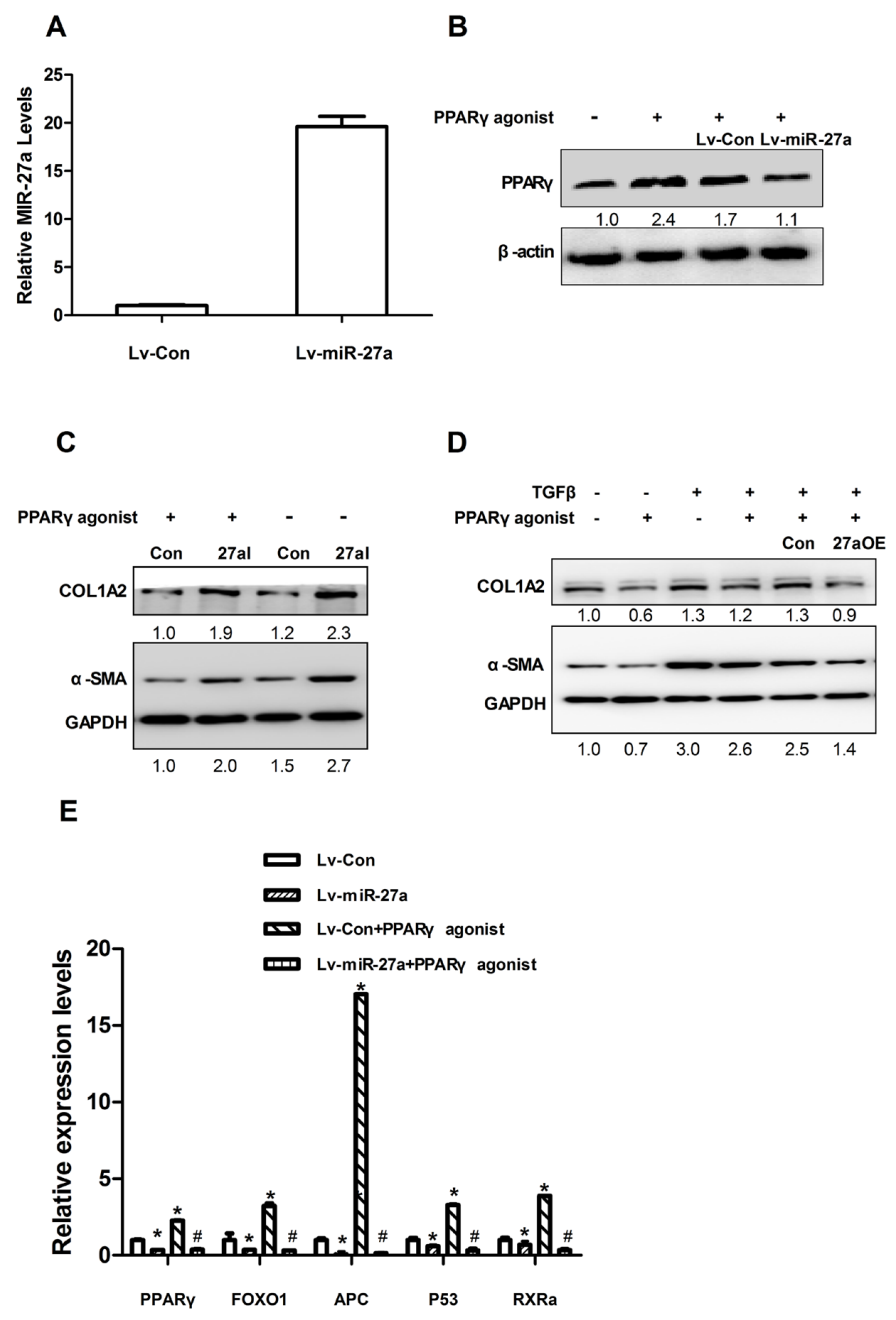
miR-27a suppresses PPARγ expression and a PPARγ agonist attenuates the effect of TGFβ in LX2 cells **(A)** The expression level of miR-27a in LX2 cells infected with Lv-miR-27a. RNU6B was used as an internal control. **(B)** LX2 cells were infected with miR-27a (Lv-miR-27a) or miRNA control (Lv-Con) for 24 h and then stimulated with 50 nM PPARγ agonist (+) or remained untreated (control, -) for 72 h before immunoblotting. **(C)** LX2 cells were transfected with miR-27a agomir (27aOE) and control agomir (Con) for 4h and then stimulated with 50 nM PPARγ agonist (+) or remained untreated (control, -) for 72 h before immunoblotting. **(D)** LX2 cells were transfected with a negative control (ConI) or miR-27a antagomir (27aI) for 4 hours, followed by stimulation with 50 nM PPARγ agonist (+) for 72 h before immunoblotting. Transfect for 48 hours, followed by stimulation with10 ng/ml TGF-β (+) or not treatment for 24 hours before immunoblotting. For immunoblotting, the intensity of each band was densitometrically quantified. The levels of target genes in each sample were normalized by that of GAPDH or β-actin (internal control). **(E)** LX2 cells were infected with miR-27a (Lv-miR-27a) or miRNA control (Lv-Con) for 24 h and then stimulated with 50 nM PPARγ agonist (+) or not treated (control, -) for 72 h before RT-qPCR. For qPCR analysis, the levels of target genes were normalized to the β-actin expression. ^*^P<0.01(vs. Lv-Con); ^#^*P*<0.01 (vs. Lv-Con+ PPARγ agonist).

We next explored the mechanisms responsible for the miR-27a-induced up-regulation of α-SMA and COL1A2 expression. To identify validated and predicted targets of miR-27a, we searched the miRTarBase and miRWalk databases, and found that miR-27a may regulate the expression levels of PPARγ, FOXO1, APC, P53, and RXRα. As expected, we found that the introduction of miR-27a repressed PPARγ expression (Figure 7B) in LX2 cells. Furthermore, similar to miR-27a inhibition, a PPARγ agonist significantly inhibited α-SMA and COL1A2 expression and attenuated the TGFβ-induced elevation of α-SMA and COL1A2 levels (Figure 7C). Interestingly, the inhibition of miR-27a increased the PPARγ-induced down-regulation of α-SMA and COL1A2 protein levels (Figure 7D).

It was previously reported that miR-27a also regulates several target genes, such as FOXO1, APC, P53 and RXRα [17–20], which are associated with liver fibrosis/cirrhosis [21–24]. We found that the levels of PPARγ, FOXO1, APC, P53 and RXRα mRNAs expression were significantly down-regulated in the tissue of rats with liver cirrhosis compared to those in normal animals (Supplementary Figure 2). We found that the overexpression of miR-27a in HSCs also led to the significant down-regulation of PPARγ, FOXO1, APC, P53, and RXRα mRNAs expression in LX2 cells (Figure 7E). Moreover, a PPARγ agonist increased the up-regulation of PPARγ, FOXO1, APC, P53, and RXRα mRNAs expression in LX2 cells (Figure 7E). The overexpression of miR-27a inhibited the PPARγ-induced up-regulation of these fibrosis-related genes (Figure 7E).

## DISCUSSION

Many miRNAs are presently in circulation. Circulating miRNAs are very stable in plasma and can be found in lipid or lipoprotein complexes [25], apoptotic bodies [26], microvesicles [27] or exosomes [28]. Recent studies have shown that the levels of circulating miRNAs can be significantly altered at different physiological stages and pathological conditions. Specific circulating miRNA profiles have been reported for various diseases [11, 13–16]. These circulating miRNA profiles have been described to correlate with differentially expressed miRNAs in diseased tissue, such as the liver following injury by drugs [29] or a related cancer [12]. Moreover, some disease-specific profiles can inform both the diagnosis and prognosis [30, 31]. These findings parallel the use of circulating miRNAs as reliable noninvasive biomarkers for disease detection.

In the present study, we focused on the serum miRNAs and expected to establish their contribution to liver cirrhosis, which results in a poor outcome and end-stage liver disease. We performed miRNAs profiling by microarrays using serum samples from HBC and CHB cases. The miRNA profile analysis showed that 38 miRNAs were differentially expressed between the HBC and CHB subjects; 33 were up-regulated, including miR-27a, miR-27b, miR-142-3p, miR-151-5p and miR-424, and 5 were down-regulated in HBC patients compared with the levels in CHB patients (fold-change>2.0 and *P*<0.01) (Table 2). Although the role of miR-27a in rat HSCs activation has been reported [20], its effects on the occurrence and development of HBC remain unclear. We focused on miR-27a to determine whether it can distinguish HBC from CHB and to identify the potential mechanisms. Additionally, miR-122 was previously described to be a liver-specific miRNA [32]. In rodents, liver injury induced by alcohol or chemicals increases the level of serum or plasma miR-122, and this increase occurs earlier than the increase in ALT, a commonly used marker [13, 33]. Moreover, the level of plasma miR-122 exhibits an excellent correlation with the necro-inflammatory activity of HBV [34] and HCV infection [35, 36]. Therefore, miR-122 was also selected for the measurement, serving as a positive control.

**Table 2:** Clinical parameters of participants

Parameters	HBC	CHB	Healthy
Individuals (n)	107	84	43
Male	61	49	23
Female	46	35	20
Age (years)	38.1±13.4	38.5±12.2	36.7±11.9
ALT (IU/L)	49.1(15-122)	73.7(14-525)	20.3(10-31)
AST (IU/L)	56.0(19-187)	52.7(16-353)	19.9(14-35)
GGT (IU/L)	43.4(14-131)	48.8(10-284)	21.3(14-27)
ALP (IU/L)	170.6(33-1206)	83.2(31-186)	55.4(42-96)
TBIL (μM/L)	35.0(16-84)	18.7(5-44.8)	14.9(9.8-20.5)
HBV DNA	39960948 (0-94800000)	14054341 (0-253200000)	0
HBV status (n)			
HBsAg +	107	84	0
HBsAg -	0	0	43
Compensation	75		
Decompensation	32		

Ages are given as mean ± S.D.; Values of alanine aminotransferase (ALT), aspartate aminotransferase (AST), Gamma-glutamyltransferase (GGT), alkaline phosphatase (ALP), total bilirubin (TBIL) and hepatitis B virus (HBV) DNA were given as medians (range); CHB: chronic hepatitis B; HBsAg: hepatitis B surface antigen.

We analyzed the expression levels of serum miR-27a and miR-122 in HBC, CHB, and Ctrl subjects by RT-qPCR for the subsequent experiments. The serum miR-27a level was significantly up-regulated in HBC, and could differentiate HBC from CHB and Ctrl subjects (*P*<0.0001 for both) (Figure 2A). Moreover, miR-27a expression was well correlated with disease progression in the HBC subjects and was significantly higher in patients with decompensated HBC than in patients with compensated HBC (*P*=0.0009) (Figure 2C). Serum miR-27a measurement may help for the diagnosis of advanced cirrhosis. Additionally, serum miR-122 was considerably higher in HBC patients than in Ctrl subjects (*P*<0.0001). Moreover, miR-122 level in CHB patients were higher than those in HBC subjects (*P*<0.0001) (Figure 2B). These results suggest that elevated miR-122 expression is an early event in the pathogenesis of HBV infection. ROC curve analyses revealed that serum miR-27a may be a useful marker for discriminating between HBC and CHB. In particular, the ROC curve analyses suggested that using both miR-27a and miR-122 (ROC=0.94) was preferable to using miR-27a (ROC=0.82) or miR-122 (ROC=0.87) alone as a marker for discriminating between HBC and CHB. Furthermore, we demonstrated the expression levels of serum miR-27a and miR-122 were markedly up-regulated in DMN-induced liver cirrhosis in rats compared to those in saline-treated rats (Figure 4A and 4B).

Studies have shown that miRNAs can be expressed in a tissue-specific or cell-specific manner [8, 10–12]. Several recent studies provided additional evidence that some miRNAs are not only passively released, but also actively secreted from cells and integrated into vesicles, and that they could potentially serve as messengers to influence gene transcription in other cells [28, 35, 36]. Chronic inflammation and the accumulation of extracellular matrix persist in liver injury in HBC, and they may lead to detectable levels of fibrosis-related miRNAs in the circulation [15]. HSCs, the major mesenchymal cell type in the liver, are well known for their critical functions in liver fibrosis, which is activated by TGFβ1. In this study, we found that miR-27a expression was significantly up-regulated in human hepatic stellate LX2 cells and culture medium activated by TGFβ1 (Figure 5A and 5B), while miR-27a did not show specific regulation changes following TGFβ1 treatment in normal human hepatic L02 and hepatocellular carcinoma HepG2 cells and the culture medium (Figure 5A and 5B). We speculated that the release of miR-27a from HSCs activated by TGFβ1 is the principal source of extracellular circulating miR-27a in HBC.

We conclude that circulating miR-27a may be a more specific predictor for HBC than miR-122. In this work, we focused mainly on the role of miR-27a in HSCs. We found that miR-27a has a moderate effect on inhibiting the proliferation of HSCs (Figure 6A). The inhibition of miR-27a attenuated the TGFβ-induced expression of fibrosis-related genes (Figure 6B). These results suggest that miR-27a up-regulation may facilitate the TGFβ-induced activation of HSCs. Emerging evidence suggests the importance of miR-27a overexpression in HSCs [20, 37], although the full molecular mechanisms have yet to be established. We found that miR-27a directly targets PPARγ and promotes the expression of profibrotic genes in LX2 cells. Previous studies have reported that miR-27a promotes renal tubulointerstitial fibrosis [38], podocyte injury [39] and mesangial cell injury [40] by suppressing PPARγ in diabetic nephropathy. MiR-27a has also been demonstrated to repress the activity of PPARγ in hepatocellular carcinoma cells [41]. These findings provide evidence that PPARγ is a critical, intermediate, downstream modulator of miR-27a. We found that miR-27a directly targeted PPARγ and promoted the expressions of profibrotic factors, such as α-SMA and COL1A2, in LX2 cells (Figure 7B and 7C). Moreover, a PPARγ agonist inhibited the expression of α-SMA and COL1A2 (Figure 7C). The overexpression of miR-27a in HSCs led to significant up-regulation of α-SMA and COL1A2 expression levels (Figure 7C). Interestingly, the inhibition of miR-27a increased the PPARγ-induced down-regulation of α-SMA and COL1A2 protein levels (Figure 7D), suggesting that miR-27a is involved in the activation induced by TGFβ1 in HSCs through the up-regulation of α-SMA and COL1A2 expression by targeting PPARγ.

We further identified, validated and predicted targets of miR-27a using the miRTarBase and miRWalk databases. MiR-27a has previously been demonstrated to regulate several target genes, such as FOXO1 [17], APC [18], P53 [19] and RXRα [20], which are associated with liver fibrosis/cirrhosis [21–24]. It has been reported that FOXO1 plays a crucial role in the transdifferentiation and the proliferation of HSCs in liver fibrosis [21]; APC, which negatively regulates Wnt signaling, is associated with liver fibrosis [22]; P53 can promote apoptosis and inhibit the proliferation of HSCs [23]; and RXRα can inhibit the HSCs proliferation and reverse the phenotype of activated HSCs [24]. We found that the mRNAs expression levels of PPARγ, FOXO1, APC, P53, and RXRα were significantly down-regulated in the tissues of rats with liver cirrhosis compared to those in normal animals (Supplementary Figure 2). Furthermore, the overexpression of miR-27a in LX2 cells also led to the significant down-regulation of PPARγ, FOXO1, APC, P53, and RXRα mRNAs expression (Figure 7E).

PPARγ is expressed in quiescent HSCs, and its expression decreases after HSCs transdifferentiation. The loss of expression of PPARγ constitutes one of the most important molecular mechanisms in HSCs activation [42, 43]. The activation of PPARγ causally stimulates p53 expression, which critically leads to cell growth arrest and senescence in activated HSCs [44]. FOXO1 plays a crucial role in the transdifferentiation and proliferation of HSCs in liver fibrosis. FOXO1-transduced HSCs showed higher PPARγ and lower TIMP-1 mRNA levels compared with those in GFP-transduced HSCs [21]. APC, which negatively regulates the β-catenin pathway, was found to be associated with liver fibrosis. Activation of the β-catenin pathway can inhibit the expression of PPARγ [45]. PPARγ forms a heterodimer with RXRα, and the complex subsequently binds to a specific DNA sequence, the peroxisome proliferating response element (PPRE), which is located in the promoter region of PPARγ target genes and modulates their transcription [46].

MiR-27a is a direct regulator of PPARγ, FOXO1, APC, p53 and RXRα expression in HSCs. PPARγ agonists promote PPARγ activation in HSCs. The overexpression of PPARγ prevents miR-27a from targeting FOXO1, APC, p53, and RXRα for their down-regulation. Therefore, PPARγ agonists increase the up-regulation of PPARγ, FOXO1, APC, p53, and RXRα mRNA expression in LX2 cells. The overexpression of miR-27a inhibited the PPARγ-induced up-regulation of these fibrosis-related genes, including PPARγ, FOXO1, APC, P53, and RXRα (Figure 7E). Elucidating the miR-27a-targets regulatory network may shed light on liver fibrogenesis and may be valuable for the development of novel diagnostic and treatment approaches for liver fibrosis. More studies are needed to further define the mechanism of miR-27a-mediated growth inhibition of HSCs.

In summary, we found that miR-27a was significantly up-regulated in the serum of HBC patients, DMN-induced rat liver cirrhosis and TGFβ1-activated HSCs. Circulating miR-27a could be a potential predictor for HBC and HSCs activation. Our findings also suggest that miR-27a plays an important role in HSCs activation by targeting multiple target genes, including PPARγ, FOXO1, APC, P53, and RXRα.

## MATERIALS AND METHODS

### Study subjects and clinical parameters

Sera collected from 107 HBC, 84 CHB and 43 Ctrl subjects were included in this study. Samples from 10 CHB patients (5 males and 5 females) and 10 HBC patients (5 males and 5 females) were analyzed by miRNA microarrays to obtain serum miRNA profiles. The miRNAs with altered levels were further verified using RT-qPCR with the samples from the remaining 97 HBC patients, 74 CHB patients and 43 Ctrl subjects. Serum Ctrl samples were randomly selected from a collection of 120 individuals who underwent an annual physical examination at Shanghai Shuguang Hospital in Shanghai, China. The HBC and CHB samples were from patients seeking treatment at Shanghai Shuguang Hospital. The diagnostic criteria for CHB followed the guidelines defined by the Chinese Society of Hepatology and Chinese Society of Infectious Diseases in 2005 [47]. The diagnosis of CHB was based on increased ALT levels (above the upper limit of the normal range) in at least two blood samples assayed over a 6-month period and the presence of detectable hepatitis B surface (HBs) antigen and (or) HBV DNA. The diagnosis standard for HBC referred to the “Chronic hepatitis B prevention and treatment guidelines.” [48]. Post-HBV infection liver cirrhosis patients (cirrhosis group) met the following criteria: (1) with HBV infection, (2) diagnosed by two experienced pathologists, and (3) if no tissue was available, diagnosis was supported by two imaging reports (ultrasound B, CT, or MRI). The clinical parameters of these patients are given in Table 2. This study was subject to approval by the Institutional Review Board of Shanghai Shuguang Hospital. An informed consent form was issued and signed by each of the participants, and the study protocol conformed to the ethical guidelines of the Declaration of Helsinki (1964).

### Serum sample collection and RNA isolation

All serum samples were separated from freshly drawn blood and stored at -80°C. RNA in the serum and cells was isolated using a mirVana PARIS kit (Ambion, Austin, TX, USA) according to the manufacturer’s protocol, which was followed by treatment with RNase-free DNase I (Promega, Madison, WI, USA) to eliminate DNA contamination.

### Serum miRNA profiling and data analysis

The profiles of serum miRNAs from 10 HBC and 10 CHB cases were generated using an Agilent Human miRNA microarray V3 (Agilent Technologies, Santa Clara, CA, USA). The microarray chip consists of 2371 different probes for a total of 851 human miRNAs. One hundred nanograms of serum RNA was used for each array. The arrays were read using an Agilent microarray scanner, and the data were extracted using Feature Extraction V10.7 (Agilent Technologies, CA). All data were transformed to log base 10. The differences between samples were calculated using unsupervised analysis (SAS system, Shanghai Biochip, Shanghai, China). Only those miRNAs with a fold-difference>2.0 and a *P*-value<0.01 were considered significant.

### Quantitative real-time RT-PCR analysis

RNA was extracted from serum and medium, and RT-qPCR was performed as previously described [13]. MiRNAs were analyzed following the manufacturer’s standard protocol using the miScript SYBR Green RT-qPCR Kit (Qiagen, Valencia, CA, USA). Hepatic cell and culture medium miR-27a levels were normalized using RNU6 and cel-miR-39 as a reference, whereas serum miR-27a was normalized to miR-24 [13]. To assess the mRNA level of related genes, total RNA extracted from the cells or tissues and first strand cDNA were synthesized using 2 μg of total RNA treated with ReverTra Ace qPCR RT Master Mix with gDNA Remover (TOYOBO, LTD, Japan) according to the manufacturer’s instructions. RT-qPCR analysis was performed in triplicate with SYBR Green PCR Master Mixture (TOYOBO, LTD, Japan) using the ABI StepOne Plus Real-Time PCR System with results normalized to β-actin expression. The ΔΔCt method was used to calculate relative expression. Primer sequences used for RT-PCR are shown in Table 3 and Supplementary Table 1.

**Table 3:** Primer sequences for RT-qPCR (Human)

Name	Sequence
PPARγ-FP	TACTGTCGGTTTCAGAAATGCC
PPARγ-RP	GTCAGCGGACTCTGGATTCAG
FOXO1-FP	GGATGTGCATTCTATGGTGTACC
FOXO1-RP	TTTCGGGATTGCTTATCTCAGAC
APC-FP	AAAATGTCCCTCCGTTCTTATGG
APC-RP	CTGAAGTTGAGCGTAATACCAGT
P53-FP	GAGGTTGGCTCTGACTGTACC
P53-RP	TCCGTCCCAGTAGATTACCAC
RXRα-FP	GGACTGCCTGATTGACAAGC
RXRα-RP	TTCAGCCCCATGTTTGCCTC
β-actin-FP	TGCGTGACATTAAGGAGAAG
β-actin-RP	GCTCGTAGCTCTTCTCCA

Abbreviations: FP: forward primer; RP: reverse primer.

### Animal model

Twenty-eight male Wistar rats (weighing 150-180 g) were used. The animals were housed in an air-conditioned room at 23±3°C with 60% relative humidity and 12-hours light/dark cycles. Two distinct models of experimental hepatic fibrosis were produced in rats; one used DMN (model group, n=14) and one used normal saline (control group, n=14). In the DMN-induced fibrosis group, the rats were intraperitoneally injected with 0.5% DMN (2ml/kg body weight) for three consecutive days per week for 4 weeks as previously described [49]. Rats were sacrificed after 4 weeks and liver tissues and sera were harvested for further analysis. Liver fibrosis status was evaluated by picrosirius red staining. Paraffin-embedded tissue sections were stained with 1% Sirius red (Sigma-Aldrich, France) dissolved in saturated picric acid for 90 min at room temperature. Sections were washed twice with 0.5% acetic acid and then dipped in 70% alcohol for 5 min, followed by 5 min in xylene. The slides were mounted and then observed with a microscope. This study conformed to “The Guide for the Care and Use of Laboratory Animals” and was approved by the Ethics Committee of Shanghai University of Traditional Chinese Medicine.

### Cells and treatment

LX2 cells (donated by Dr. Xu L [50]), L02 cells and HepG2 cells were maintained in plastic culture plates in DMEM (Sigma Chemical Co., St Louis, MO, USA) supplemented with 10% fetal bovine serum (FBS) (Invitrogen, Carlsbad, CA, USA). The L02 and HepG2 cell lines were purchased from the Cell Bank of Type Culture Collection (Chinese Academy of Sciences, Shanghai, China). LX2, L02 and HepG2 cells were seeded in 6-well plates in DMEM supplemented with 0% FBS. The cells were cultured for 24 hours, and the medium was replaced with DMEM supplemented with TGFβ1 (R&D Systems, 10 ng/ml). The cultures were continued for an additional 24 hours.

### Cell transfection and infection

The miR-27a antagomir, or a negative control of the antagomir (GenePharma, Shanghai, China) were transiently transfected into LX2 cells using Lipofectamine 3000 transfection reagent (Thermo Fisher Scientific) according to the manufacturer’s protocol. The final concentration of the miR-27a antagomir or agomir and negative control of the antagomir or agomir was 100 nM. Lentiviral miR-27a (Lv-miR-27a) and empty lentiviral (Lv-Con) vectors were constructed by Genechem Company (Shanghai, China), and they were infected into LX2 cells according to the manufacturer’s instructions. The cells were cultured for 24 hours, and the medium was replaced with DMEM supplemented with a PPARγ agonist (R&D Systems, 50 μM). The cells in each group were treated for 72 hours and then harvested for further analyses.

### Cell proliferation assay

Cell proliferation was assessed with the MTS assay, which was performed according to the manufacturer’s instructions. In brief, LX2 cells were cultured at a concentration of 3^*^10^3^ cells/well in plastic plates for 24 hours and then transfected with the miR-27a antagomir, or negative controls at a final concentration of 100 nM. Before measuring LX2 cell proliferation, the medium was changed for another 24-72 hours. Then, 20 μl of the CellTiter AQ Solution (Promega, Madison, WI), which contained MTS, was added to each well. After 4 hours of incubation at 37°C, the absorbance at 490 nm was measured.

### Western blot analysis

Total protein were extracted from cells with a protein extraction reagent (Thermo Fisher Scientific, USA). Membranes were exposed to a rabbit anti-PPARγ antibody (Santa Cruz, sc-7196, diluted 1:100), rabbit anti-COL1A2 (Abcam, ab96723, diluted 1:1000) or rabbit anti-α-SMA antibody (Abcam, ab5694, diluted 1:500) or a rabbit β-actin or GAPDH antibody (Cell Signaling Technology, 4970S, 2118S, diluted 1:2000). After incubation with the HRP-conjugated secondary antibody, blots were visualized with the Pierce ECL Western Blotting Substrate (Thermo Fisher Scientific, USA). The relative protein expression levels were normalized to the β-actin or GAPDH levels.

### MiRNA target prediction

To identify the validated and predicted targets of miR-27a, we searched the miRTarBase (http://miRTarBase.mbc.nctu.edu.tw/) and miRWalk (http://zmf.umm.uni-heidelberg.de/apps/zmf/mirwalk/micrornapredictedtarget.html) databases.

### Statistical analysis

Comparisons between groups were analyzed using the Mann-Whitney *U*-test or Student’s *t* test where appropriate. Receiver-operator characteristic (ROC) curves were established to evaluate the differences in the levels of serum miRNAs between HBC, CHB, and Ctrl subjects. All tests were two-tailed, and *P*<0.05 was considered statistically significant.

## SUPPLEMENTARY MATERIALS FIGURES AND TABLE


