# Supplementary material for: MiR-27a as a predictor for the activation of hepatic stellate cells and hepatitis B virus-induced liver cirrhosis
Source: Oncotarget. 2017 Dec 15;9(1):1075–90. doi: 10.18632/oncotarget.23262 (PMC5787420; doi:10.18632/oncotarget.23262)
Supplement: Supplementary file 1 [file oncotarget-09-1075-s001.pdf]

MiR-27a as a predictor for the activation of hepatic stellate cells and hepatitis B virus-induced liver cirrhosis

SUPPLEMENTARY MATERIALS

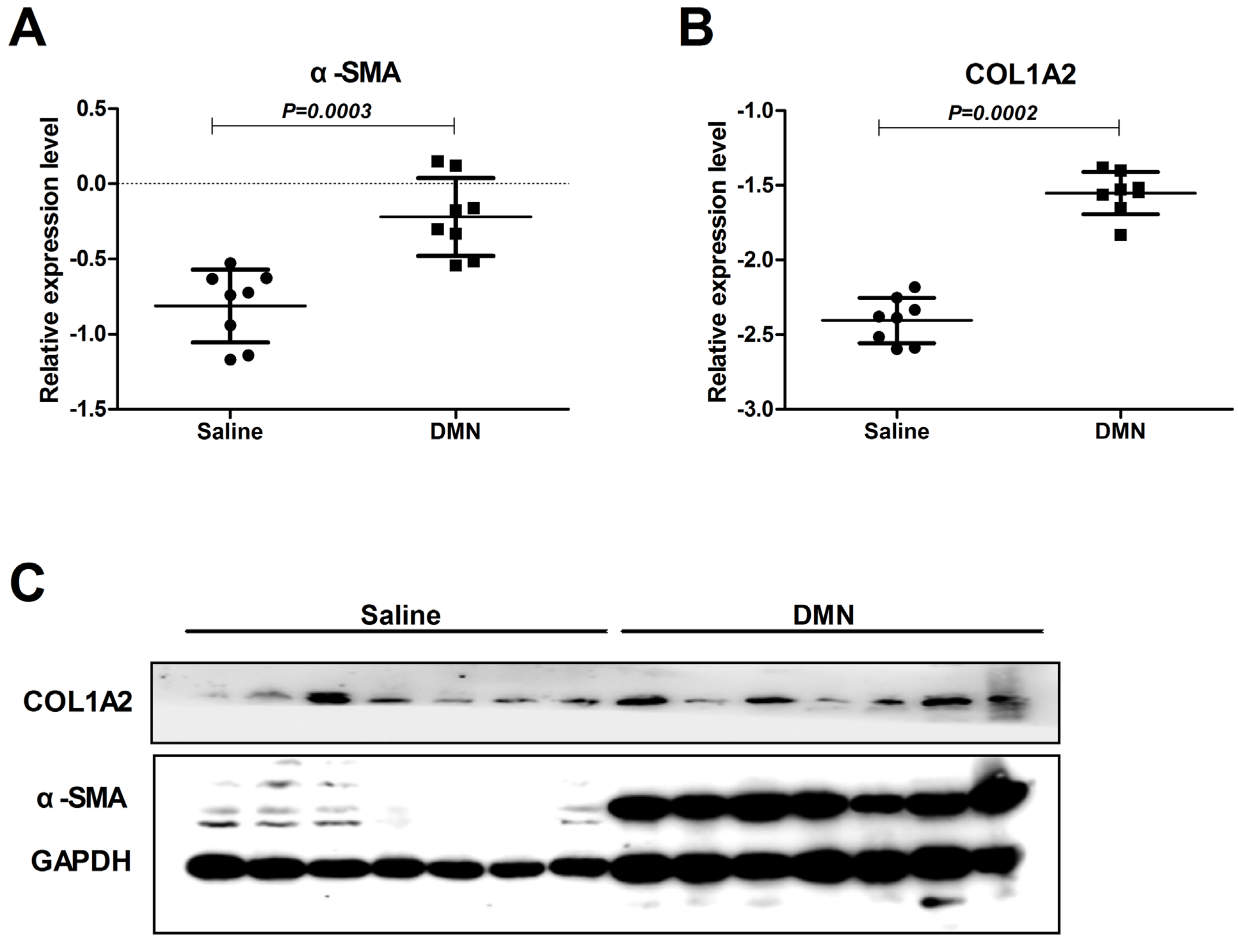

Supplementary Figure 1: The mRNA and protein levels of α-SMA and COL1A2 in a rat model of liver cirrhosis induced by DMN and saline. Total mRNA and protein extracts from liver tissues induced by DMN and saline were subjected to RT-qPCR (n=8, respectively) for α-SMA **(A)**, and COL1A2 **(B)** or immunoblotting **(C)** (n=7, respectively). GAPDH was used as an internal control.

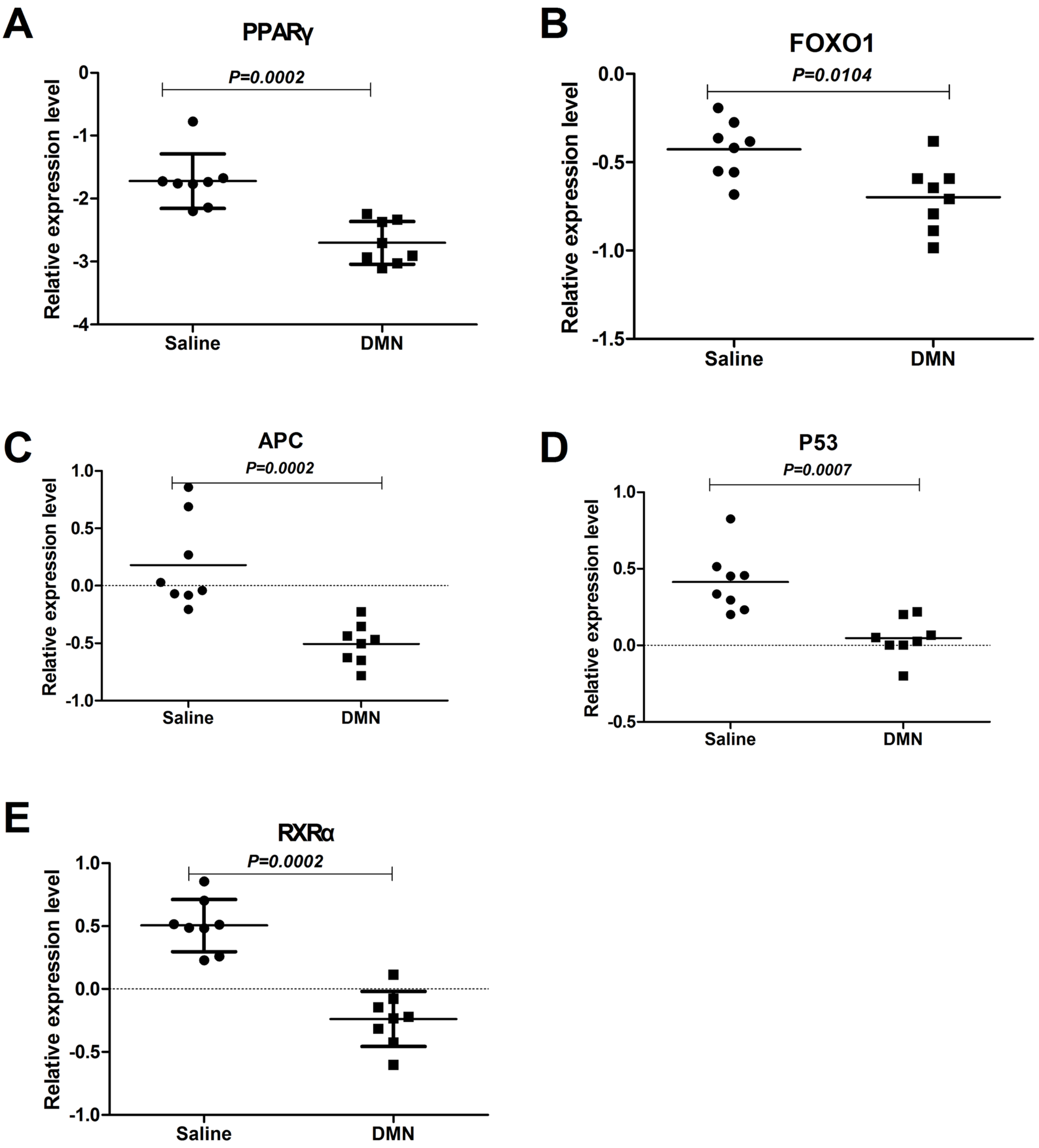

Supplementary Figure 2: The mRNA levels of PPARγ, FOXO1, APC, P53, and RXRα in a rat model of liver cirrhosis induced by DMN and saline. Total mRNA extracts from liver tissues induced by DMN and saline were subjected to RT-qPCR (n=8, respectively) for PPARγ **(A)**, FOXO1 **(B)**, APC **(C)**, P53 **(D)**, and RXRα **(E)**. *GAPDH* was used as an internal control.

Supplementary Table 1: Primer sequences for RT-qPCR (Rat)

Name	Sequence
PPARγ-FP	CATGGTGCCTTCGCTGATG
PPARγ-RP	GTCAGCGGACTCTGGATTCAG
FOXO1-FP	CAGCCAGGCACCTCATAACA
FOXO1-RP	TCAAGCGGTTCATGGCAGAT
APC-FP	TAGGAAAAGCAGCGCAGACA
APC-RP	GGACTCTGGGCTCCACTAGA
P53-FP	GATAGTACTCGGCCCCCTCT
P53-RP	GTAGGTGCCAGGTCCAACAA
RXRα-FP	CAGGTGAACTCTTCATCCCTGA
RXRα-RP	AACTCAGGGTGCTGATAGGC
COL1A2-FP	TGGTCAGAGCAGTGTGCAAT
COL1A2-RP	CACTGCAAACGTTTTGGGGA
α-SMA-FP	GTCCCAGACATCAGGGAGTAA
α-SMA-RP	TCGGATACTTCAGCGTCAGGA
GAPDH-FP	GTCTTCACTACCATGGAGAAGG
GAPDH-RP	TCATGGATGACCTTGGCCAG

Abbreviations: FP: forward primer; RP: reverse primer.
